# Combining the DNA Repair Inhibitor Dbait With Radiotherapy for the Treatment of High Grade Glioma: Efficacy and Protein Biomarkers of Resistance in Preclinical Models

**DOI:** 10.3389/fonc.2019.00549

**Published:** 2019-06-19

**Authors:** Julian Biau, Emmanuel Chautard, Nathalie Berthault, Leanne de Koning, Frank Court, Bruno Pereira, Pierre Verrelle, Marie Dutreix

**Affiliations:** ^1^Centre de Recherche, Institut Curie, PSL Research University, Paris, France; ^2^UMR3347, CNRS, Orsay, France; ^3^U1021, INSERM, Orsay, France; ^4^Research Department, Université Paris Sud, Orsay, France; ^5^INSERM, U1240 IMoST, Université Clermont Auvergne, Clermont Ferrand, France; ^6^Radiotherapy Department, Centre Jean Perrin, Université Clermont Auvergne, Clermont-Ferrand, France; ^7^Pathology Department, Centre Jean Perrin, Université Clermont Auvergne, Clermont-Ferrand, France; ^8^Laboratory of Proteomic Mass Spectrometry, Centre de Recherche, Institut Curie, Paris, France; ^9^Department of Translational Research, Institut Curie, PSL Research University, Paris, France; ^10^GReD Laboratory, CNRS UMR 6293, INSERM U1103, Université Clermont Auvergne, Clermont-Ferrand, France; ^11^Biostatistics Department, DRCI, Clermont-Ferrand Hospital, Clermont-Ferrand, France; ^12^U1196, INSERM, UMR9187, CNRS, Orsay, France; ^13^Radiotherapy Department, Institut Curie Hospital, Paris, France

**Keywords:** radiation therapy, high grade glioma, Dbait, preclinical study, double-strand break, single-strand break, radioresistance

## Abstract

High grade glioma relapses occur often within the irradiated volume mostly due to a high resistance to radiation therapy (RT). Dbait (which stands for DNA strand break bait) molecules mimic DSBs and trap DNA repair proteins, thereby inhibiting repair of DNA damage induced by RT. Here we evaluate the potential of Dbait to sensitize high grade glioma to RT. First, we demonstrated the radiosensitizer properties of Dbait in 6/9 tested cell lines. Then, we performed animal studies using six cell derived xenograft and five patient derived xenograft models, to show the clinical potential and applicability of combined Dbait+RT treatment for human high grade glioma. Using a RPPA approach, we showed that Phospho-H2AX/H2AX and Phospho-NBS1/NBS1 were predictive of Dbait efficacy in xenograft models. Our results provide the preclinical proof of concept that combining RT with Dbait inhibition of DNA repair could be of benefit to patients with high grade glioma.

## Introduction

High grade gliomas are the most frequent primary brain tumors in adults (1, 2). They represent an important source of morbidity and mortality and are a public health care challenge (3, 4). Maximal possible surgery is generally the first step of the management of high grade gliomas. Radiotherapy (RT) (+/- chemotherapy), is a major adjuvant therapy that improves survival (5, 6). Despite these treatments, median survival remains very low (1, 4). Early recurrence often occurs in the irradiated volume due to a high radioresistance of glioblastoma cells (7–10). These recurrences emphasize the need to overcome tumor radioresistance with new molecules that target pathways underlying the mechanisms of such resistance (10–12).

**Figure 1:**
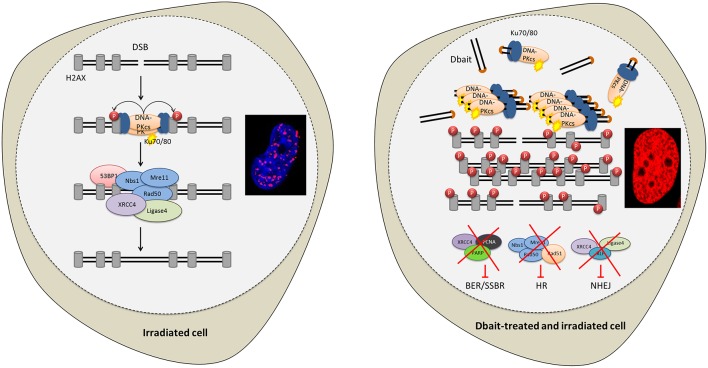
Disrupting DNA repair with Dbait molecules. Radiation and Dbait treatment induce DNA repair signaling disturbance. Dbait recognize and activates DNA-PK complex leading to its activation and its subsequent nuclear targets phosphorylation visualized by pan-nuclear γ-H2AX. When a DSB occurs in the DNA, the DNA damage signaling system activated by Dbait is spread across modified chromatin and prevents the arrival of proteins involved in DSB repair at site of the damage. Consequently, both non-homologous end joining (NHEJ) and homologous recombination (HR) were inhibited. Moreover, Dbait can also recognize PARP (mainly involved in BER and SSBR) causing its autoPARylation and leading to several BER and SSBR proteins recruitment on Dbait molecules. All these factors are thus hijacked far from the DNA damage site causing to BER/SSBR inhibition.

The cytotoxicity of RT is mostly due to DNA damage (13). About 10,000 damaged bases, 1,000 single-strand breaks (SSB) and 40 double-strand breaks (DSB) are produced per gray, and per cell (13, 14). The most severe RT-induced DNA damages are DSB that are lethal to the cell if not repaired (15). The capacity of cancer cells to recognize DNA damages and initiate repair plays a major role in radioresistance (16–18). DNA repair inhibition could make cancer cells particularly sensitive to the DNA damaging treatments like RT (18, 19). Therefore, to inhibit DNA repair, we designed innovative molecules called Dbait (for DNA strand break bait). Dbait are 32 base-pair deoxyribonucleotides forming intramolecular DNA double helix mimicking DNA damages (18, 20–22). They act as a bait for DNA damage signaling enzymes inducing a “false” DNA damage signal that prevents repair enzyme recruitment at damage site and ultimately inhibits DSB and SSB repair pathways (Figure 1) (18, 20–22). Dbait was tested in combination with RT in first-in-human phase 1 clinical trial for the treatment of skin metastases of melanoma with encouraging results (23).

To decipher the mechanisms sustaining resistance to anticancer treatments is one of the most current challenges to avoid treatment escape. High-throughput screening strategies are widely used for the identification of predictive and prognostic biomarkers (24, 25). The most currently used analyzed the RNA content (transcriptome) or DNA modification (genome). However, in mammalian, it is widely accepted that regulatory modifications occur at the protein levels (25–27). Therefore, to explore *in vivo* predictive biomarkers of RT efficacy we used reverse-phase protein array (RPPA), a technology using high-throughput antibody-based detection. It requires just a few micrograms of protein lysate and allows measuring protein expression and their main modification in a highly quantitative manner (25, 27, 28). Hundreds of samples can be analyzed simultaneously and thus generate large datasets to identify potential biomarkers (25, 29).

In this preclinical study, we analyzed the potential of Dbait to sensitize high grade glioma to RT. First, we demonstrated the radiosensitizer properties of Dbait *in vitro*. Secondly, animal studies were performed to test the clinical potential of the combination of Dbait and RT for the treatment of high grade glioma. We identified potential protein biomarkers of resistance using RPPA. For that purpose, we assayed a selection of proteins and modifications involved in different RT signaling pathways.

## Materials and Methods

### Cell Culture and Dbait Molecules

Nine human high grade glioma cell lines were used (CB193, MO59J, MO59K, SF763, SF767, SNB19, T98G, U87MG, and U118MG) and were grown using a 10% Fetal Calf Serum DMEM medium in a humidified incubator containing 5% CO2 at 37°C as previously described (25).

As already described (18): Dbait molecules consist in 32 base-pair oligonucleotides (5'-GCTGTGCCCACAACCCAGCAAACAAGCCTAGA-(H)-TCTAGGCTTGTTTGCTGG GTTGTGGGCACAGC-3', Eurogentec, Seraing, Belgium). A short inactive molecule, Dbait-8H (5'-ACGCACGG-(H)-CCGTGCGT-3') was used as control in the *in vitro* experiments. H is a hexaethyleneglycol linker and the letters underlined indicate the phosphorodiamidate nucleosides.

### *In vitro* Dbait and Irradiation Treatments and Cell Survival Assay

Dbait (1.25 mg.L^−1^) or transfection control, complexed with 11 kDa polyethylenimine (PEI) (Polypus-transfection, Illkirch, France) were used to treat the cells as previously published (18, 21). Cells were incubated during 5 h for transfection in serum-free RPMI medium (in twenty-four-well plates). After transfection, the medium was removed and replaced with complete DMEM (Gibco, Cergy Pontoise, France) (18). Cells were then subjected to 2.5-Gy irradiation, using a ^137^Cs unit (0.5 Gy/min). Nine days later, cell fixation (paraformaldehyde 4%) and permeabilization (Triton X100 0.5%) were done, and the number of nuclei was estimated following staining with TO-PRO3 for 10 min. Nuclear staining signals were determined by imaging with an infrared scanner (LI-COR Odyssey).

### Western Blot

Cells were harvested and boiled 10 min in Laemmli buffer and subjected to SDSPAGE. Proteins were transferred to nitrocellulose membranes, blocked by incubation (1 h) with Odyssey buffer (LI-COR Biosciences, Lincoln, NE, USA). Membranes were incubated overnight at 4°C with primary antibody diluted in Odyssey buffer. Depending on primary antibodies, the membranes were then probed with goat secondary antibodies (anti-mouse or anti-rabbit) conjugated to Alexa Fluor 680 (Invitrogen) or IRdye 800 (Rockland Immunochemicals, Gilbertsville, PA, USA). Anti-γ-H2AX (Upstate, Millipore, Molsheim, France) and anti-β-actin clone AC-15 mouse monoclonal antibodies (Sigma-Aldrich, Saint-Quentin-Fallavier, France) were used. The obtained signals were analyzed with the Odyssey Infrared Imaging System (LI-COR Biosciences) and Odyssey software.

### Dbait and Irradiation Treatments in Mice

Xenografts derived from cell lines (CDX) and patient derived xenograft (PDX; ODA-17GIR, GBM-1-HAM, GBM-14-RAV, GBM-14-CHA, ODA-4-GEN) were, respectively obtained by injecting 4 × 10^6^ cells of each cell line into the flank, and by successive grafting into scapular area of adult female nude mice (Swiss nu/nu, 6–8 weeks, Janvier, Le Genest Saint Isle, France) (10). Small fragments of PDX tumors were grafted subcutaneously into the flank of nude mice before experiments. When the tumor volume were around 125 mm^3^, mice were divided into uniform groups (*n* = 6 to 12) (18): no treatment (*NT*), RT alone for 2 weeks (*R*T_2*w*_: 6x5Gy), Dbait alone for 2 weeks (*Dbait*: 6x3nmol) and RT + Dbait for 2 weeks (*R*T_2*w*_+*Dbait* 6x5Gy+6x3nmol). We had previously checked that mock treated animals did not show any change in tumor growth or survival as compared to animals only treated with or without RT (21). In the same way as beforehand (18), Dbait molecules with *in vivo*-jet polyethylenimine (PEI) reagent (Polyplus Transfection) at the N/P ratio 6 were diluted in 100 μL of 5% glucose. Dbait was combined with PEI to facilitate cellular delivery (21). Prior to injection, the Dbait-PEI mixture was incubated for 15 min at room temperature. Dbait intratumoral injections were realized 5 h before each RT session. To deliver RT by a ^137^Cs unit (0.5 Gy/min), a shield was conceived to spare about two-thirds of the animal's body. Doses were measured by thermoluminescence dosimetry. Tumors were monitored for all experiments with a digital caliper every 2–3 days. The formula (length × width × width/2) was used to calculate the tumor volumes. Mice weight was determined every week and followed up for 200 days. When tumors attained 2000 mm^3^, animals were sacrificed according to ethical recommendations. All animals were housed in our animal facility, and all experiments were approved by the Local Committee on Ethics of Animal Experimentation.

### Immunofluorescence Staining and Dog MRI

The MRI of a boxer dog having spontaneously developed a brain tumor was performed at the Veterinary School of Maisont-Alfort (94-France) by Dr. P. Devauchelle and tumor samples were obtained with the consent of the dog owner. For immunofluorescence staining, cells were processed as previously described (20, 30). Microscopy was performed at room temperature with the Leica SP5 confocal system, attached to a DMI6000 stand, with a 636/1.4 oil immersion objective. Images were processed with the freely available ImageJ software (http://rsb.info.nih.gov.gate1.inist.fr/ij/) and the Leica SP5 confocal system.

### Antibodies and Validation for RPPA

We explored 39 total proteins, 26 phosphoproteins and then calculated 23 ratios of phosphoproteins on total proteins giving a total of 88 protein biomarkers (Table S1) involved in 10 different signaling pathways: tyrosine kinase signaling, SAPK/JNK signaling, stress signaling, DNA repair, PI3K pathway, apoptosis, cell cycle, adhesion/cytoskeleton, MAPK/ERK signaling and NFκB signaling. As reported earlier, before being used in RPPA, the antibodies quality and specificity were confirmed by Western blotting on a large panel of cell lines (25).

### Reverse Phase Protein Array (RPPA)

Proteins from 11 subcutaneous xenograft models were analyzed (6 replicates with 2 different locations in three different tumors per model). Tumors were mechanically dissociated (10) and protein concentration was determined using the Reducing Agent Compatible BCA kit (Pierce, Rockford, USA). The samples were then processed using previously reported method (10). Briefly, serial dilutions of samples (from 2 to 0.125 mg/ml) were placed on nitrocellulose-covered slides (2470 Arrayer, Aushon Biosystems, Billerica, MA) before incubation overnight at 4 °C with specific antibodies. Slides were then probed with horseradish peroxidase-coupled secondary antibodies (Jackson ImmunoResearch, Newmarket, UK) for 1 h at room temperature. After an amplification step, the arrays were probed with Cy5-streptavidin (Jackson ImmunoResearch) for 1 h at room temperature. Finally, the processed slides were scanned with a GenePix 4000B microarray scanner (Molecular Devices, Sunnyvale, CA) and Spot intensity was evaluated with MicroVigene 4.0.0.0 software (VigeneTech Inc., Carlisle, MA). Quantification of the data was done with SuperCurve (31), and the data were normalized against negative control slides and Sypro Ruby slides.

### Statistical Analysis

Data analysis was realized with R v2.15.1 (http://www.cran.r-project.org). The tests were two-sided, with a Type I error set at α = 0.05. To explore variations between groups, Mann-Whitney tests were done according to sample size, and if assumptions of parametric test are not met (normality and homoscedasticity). The Kaplan–Meier method was used to draw the survival curves. The log-rank test was used to compare the survival fraction of groups (NT: not treated, RT or RT+Dbait). *P* ≤ 0.05 was considered to be a significant difference.

## Results

### Dbait Disorganizes Repair of Radio-Induced DNA Damage in High Grade Glioma Cell Lines and Leads Proliferation Inhibition

**Figure 2:**
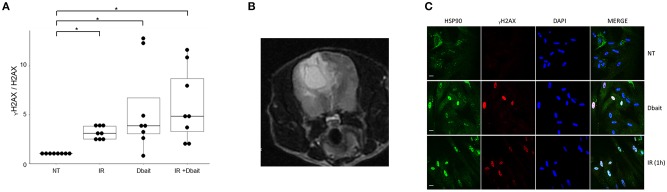
_⋎_H2AX induction in gliomas cells after Dbait treatment. **(A)**_⋎_H2AX induction in human gliomas cell lines after Dbait treatment. The nine glioma cell lines were untreated (NT), irradiated (IR, 10Gy), treated with Dbait (5h) or treated with Dbait and irradiated. One hour after treatment completion, total proteins were electrophoresed followed by immunobloting. The blots were analyzed using the Odyssey Infrared Imaging System (LI-COR Biosciences) and Odyssey software. The induction of _⋎_H2AX (_⋎_H2AX on H2AX ratio) is presented. Mann-Whitney test was performed (*p < 0.05). **(B)** MRI of a boxer dog with a spontaneously brain tumor. **(C)** Activation of DNA damage response in dog glioblastoma. Dissociated cells of dog glioblastoma were untreated (NT), irradiated or treated with Dbait (5 h). Cells were fixed and permeabilized after treatment before the use of anti- _⋎_H2AX, anti-HSP90 antibodies and DAPI.

In a previous study, we have shown that Dbait lead to activation of the DNA-dependent protein kinase (DNA-PK) (20). This hyperactivation triggers phosphorylation of H2AX and other markers such as RPA32, CHK2 and HSP90 (20, 30), prevents detection of the radio-induced DSBs and further recruitment of DNA repair enzymes at damage site (Figure 1). First, we tested the potential of Dbait to induce DNA-PK activation in human glioblastoma cell lines by assaying phosphorylated H2AX proteins by Western blot in the 9 high grade glioma cell lines. Western blot analysis showed that in all the glioma cell lines except in the DNA-PK deficient MO59J cell line, Dbait induced phosphorylation of H2AX (Figure 2A). As already published (20) Dbait induced phosphorylation of H2AX is strictly dependent of DNA-PKcs kinase activity. In contrast, irradiation induced γ-H2AX foci that are mainly due to ATM activation, in all cell lines including MO59J. Combining Dbait and irradiation induced equal to superior level of γ-H2AX. The level of H2AX was not significantly affected by the various treatments (Supplementary Figure 1). As we had access to samples from a dog that spontaneously developed a glioblastoma (Figure 2B), we confirmed that Dbait induced phosphorylation of both H2AX and HSP90 in dissociated cells from the brain tumor (Figure 2C). As previously observed, γ-H2AX formed foci after irradiation, at location of radio-induced DNA DSB in irradiated cells whereas it distributed all over the chromatin after Dbait treatment (in at least 65% of the cells), showing DNA-PK activation in absence of chromosome damage (20).

**Figure 3:**
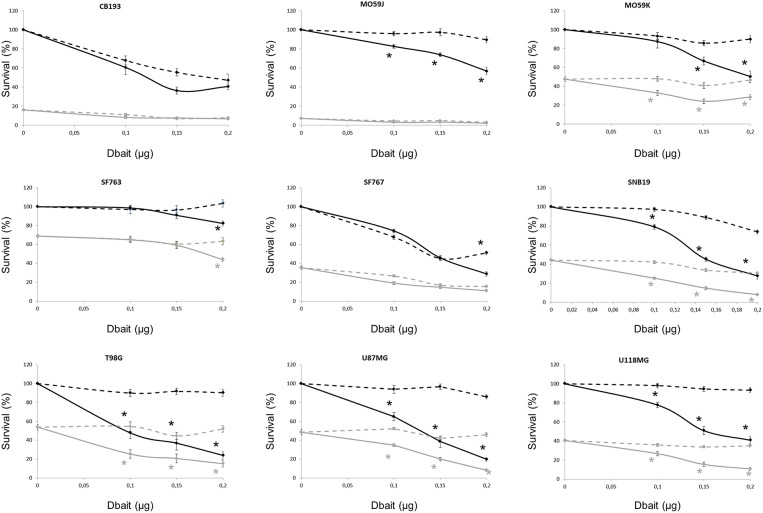
Dbait impact on cell survival. Cell survival assay of high grade glioma cell lines were treated with Dbait (solid line) or control (dotted line). Cells were irradiated at 2.5Gy (gray line) or not (black line) 5 h after Dbait treatment. Data are represented as mean values ± standard error. Mann-Whitney test was performed (*p < 0.05).

The consequences of DNA-PK hyperactivation for cell survival after irradiation were investigated. Nine high-grade glioma cell lines were treated with Dbait or control (Dbait-8h) 5 h before RT to allow DNA-PK activation before inducing damage (Figure 3). As we have previously reported (18), without RT, Dbait treatment itself was able to decrease cell survival. For 6/9 cell lines (MO59K, SF763, SNB19, U118MG, U87MG, and T98G), the combination of Dbait and RT led to a significant radiosensitization (*p* < 0.05). SF763 was sensitized only at the highest dose of Dbait. For 3/9 cell lines (CB193, MO59J and SF767), radiosensitization was not statistically significant. γ-H2AX increase after Dbait treatment was observed in SF767 (Figure 2) eliminating the possibility that the lack of sensitization could be due to defect in transfection or incapacity to activate DNA-PKcs as it is the case of MO59J cells.

### Radiosensitizing Effect of Intratumoral Injections of Dbait *in vivo*

**Figure 4:**
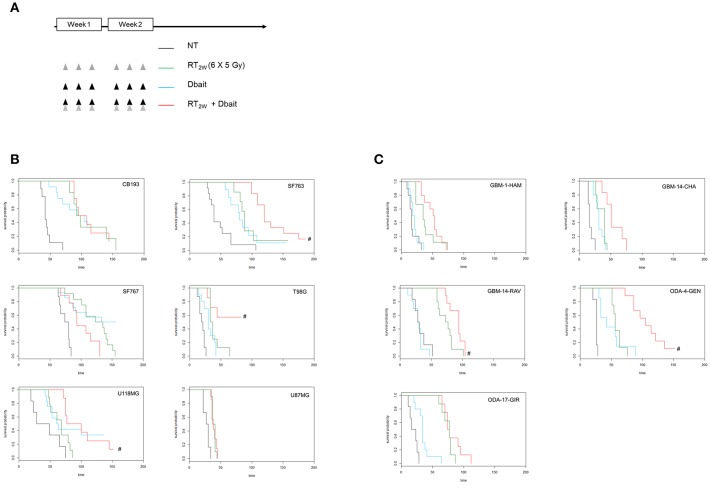
Effect of Dbait combined with radiation *in vivo*. **(A)** Xenograft models were treated with hypofractionated radiotherapy (RT; 6x5Gy in 2 weeks; green), Dbait (6 local administrations in 2 weeks; blue) or a combination of Dbait and RT (red) or untreated (black). For Cell lines Derived Xenografts (CDX, **B**) and for Patient Derived xenograft (PDX, **C**) the survival curves of groups were plotted according to the Kaplan–Meier method. The survival fraction of groups was compared using log-rank test. #*p* < 0.05 between RT_2w_ and RT_2w_+Dbait.

We have recently shown that protein status are well conserved between cell lines and tumors formed by xenografting of these cell lines (25). However, micro-environment plays an important role in tumor cell response to treatment and could modify therapy response. Therefore, we reproduced our survival analysis *in vivo* using athymic nude mice bearing glioma CDX obtained by grafting the cell lines characterized *in vitro*. Among the 9 cell lines tested *in vitro*, only six models form tumors with enough efficacy and homogeneity to allow *in vivo* treatment efficiency study. Consistent with one of the currently used stereotactic RT protocols for the reirradiation of high grade glioma (32), 6 fractions of 5 Gy were given locally over a 2 weeks period. Dbait was administered locally 5 h prior to RT, every other day (for a total of 6 sessions; Figure 4A). The combined treatment (RT_2w_+Dbait) significantly decreased tumor growth and enhanced survival of 3/6 models (Figure 4B). The survival enhancement by addition of Dbait to radiotherapy in U118MG, SF763 and T98G was, respectively of 129, 136 and 234%. SF763 which was sensitized only at the highest dose of Dbait *in vitro* appeared to be sensitive to Dbait addition to radiotherapy *in vivo*. The CB193 and SF767 models were not radiosensitized consistently with *in vitro* results. Dbait effect did not depend upon the tumor growth speed. While U87MG cells were radiosensitized *in vitro* (Figure 3), addition of Dbait to radiotherapy had no impact on survival of U87-MG *in vivo* models.

In order to confirm that the Dbait effect is not a specificity of CDX models we performed in parallel a similar analysis on five PDX directly derived from patient samples (Figure 4C). Three were radiosensitized by Dbait with increase in survival compared to RT alone of 125% for GBM-14-RAV, 128% for GBM-14-CHA and 188% for ODA-4-GEN. The two other models (ODA-17-GIR and GBM-1-HAM) were not radiosensitized by such a treatment. Interestingly, in all the treated animal models, no significant skin toxicity was observed in irradiated and Dbait-treated healthy tissue. Depending on *in vivo* model, tumor growth after Dbait treatment alone, was at the best very similar to those observed following RT alone, making the combination a better option in most of the cases.

### Predictive Biomarkers of Dbait Efficacy

**Figure 5:**
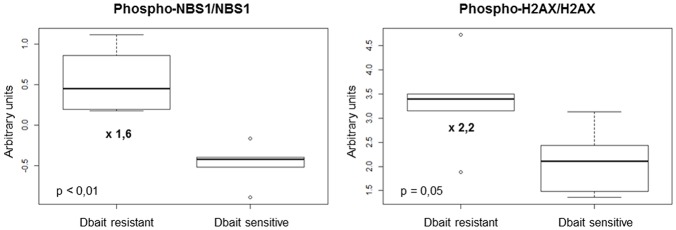
Markers involved in Dbait treatment resistance. We analyzed a total of 88 proteic markers (see Table S1) to explore tyrosine kinase signaling, SAPK/JNK signaling, stress signaling, DNA repair, PI3K pathway, apoptosis, cell cycle, adhesion/cytoskeleton, MAPK/ERK signaling and NFκB signaling. Data obtained for 11 models (without any treatment) were analyzed with 6 replicates for each xenograft models to identify makers that can predict resistance to Dbait treatment. A Mann-Whitney test was performed (*p* < 0.05) between the two groups of models (sensitive and resistant to Dbait treatment).

We then used a RPPA approach to identify protein biomarkers predictive of Dbait response of the 6 CDX and 5 PDX to RT+Dbait. Eighty-eight protein markers were analyzed: 39 total proteins, 26 phosphoproteins and 23 ratios of phosphoproteins/total proteins were analyzed. A Mann-Whitney test was performed between radiosensitized and not radiosensitized xenografts. We identified 2/88 protein biomarkers predictive of Dbait efficacy: the two most significant biomarkers were the ratio of phosphorylated forms on native forms of the two repair proteins NBS1 and H2AX. Actually Phospho-H2AX/H2AX (*p* = 0.05, fold change = 2.2) and Phospho-NBS1/NBS1 (*p* < 0.01, fold change = 1.6) were significantly higher in the xenografts that were not radiosensitized (Figure 5). Interestingly, Phospho-H2AX was not sufficient (*p* = 0.66) to predict sensitivity to Dbait radiosensitizing effect. The total amount of H2AX, which has been shown to vary extensively between cell lines (Figure 1) and tumors was also not predictive of the Dbait radiosensitization (*p* = 0.66) however their ratio became highly indicative (*p* = 0.05).

## Discussion

The resistance of cancer cells to RT is increased by efficient DNA repair activity (18, 33, 34). In the past years, many DNA repair inhibitors have been developed (18, 35–39). These strategies are mainly based on specific target inhibition. They may be overpassed by target mutation or activation of another repair pathway. On the other hand, Dbait is not a specific enzyme inhibitor. It represents a new drug strategy targeting the whole DNA DSB repair system via perturbation of DNA repair signaling (18, 20, 21). On the one side, the DNA DSB signaling system induced by Dbait is dispersed all over the chromatin and inhibits the recruitment of the DSB repair proteins at damage site. On the other side, Dbait molecules can also be recognized by PARP [major protein involved in base excision repair (BER) and single strand break repair (SSBR)]. This leads to its autoPARylation which allows the recruitment of various BER and SSBR proteins on Dbait molecules inducing BER/SSBR inhibition (18, 40).

In the present study, Dbait molecules were used to radiosensitize human high grade glioma. The experimental design was planned to assay the clinical relevance of a current hypofractionated stereotactic RT protocol used for high grade glioma reirradiation (32) and local administration of Dbait. Hypofractionated stereotactic RT is particularly interesting due to its ability to precisely deliver high doses of RT to a specific target volume in a low numbers of fractions and to spare surrounding organs at risk. Hypofractionated stereotactic RT appeared to be associated with acceptable toxicity if certain limits were observed in terms of treated volume and radiation dose (41–45). In these series, median survival was low (about 7–13 months from time of salvage treatment) suggesting a therapeutic effect in selected patients. Despite modern developments in spatial targeting, long term control of diseases is not achieved, emphasizing the need to overcome tumor radioresistance with innovative agents (10). Combined Dbait and hypofractionated stereotactic RT treatment is addressing this major challenge, and is particularly attractive to treat recurrent high grade glioma as it provides a double targeting through molecular pathway by Dbait and highly focalized ionizing radiation beam by hypofractionated stereotactic RT. This should achieve a better local control which is the main clinical objective for high grade glioma.

In this study, we used local administration of Dbait, which might limit clinical transfer in this specific indication. However, local administration to high grade glioma of different molecules has already been studied. For example, Gliadel wafer containing carmustine (BCNU) as an interstitial chemotherapy treatment is already approved for malignant glioma (46). Other modalities of local delivery such as convection-enhanced delivery have also shown preclinical and clinical promising results (22, 47, 48). Dbait distribution to the brain has already been evaluated in an RG2 rat glioma model and showed promising results (22).

One of the drawbacks of our preclinical study is that we chose to use flank xenografts rather than intracranial orthotopic xenografts (49–51). We preferred flank xenografts in this preclinical study for different reasons: the number of models and conditions tested (over 300 mice); the use of a 137Cs unit (0.5 Gy/min) which did not allow focal cerebral irradiation (necessary for 6x5Gy delivery); the need of repeated Dbait intratumoral injections; and difficulties in rigorous tumor monitoring with orthotopic xenografts by repeated imaging with high number of animals. Despite the above-cited advantages of flank xenograft models, the drawbacks include: a different microenvironment as it would be within the brain; and a lack of blood brain barrier that can alter the pharmaceutical kinetics (49–51). If the lack of blood brain barrier is not a major issue in our setting as we studied direct intratumoral injections of Dbait, the different microenvironment might significantly influence the results (52). In the past years, most documented resistance mechanisms involve secondary pathway mutations or bypass mechanisms within the tumor cells. However, the recent identification of mechanisms of therapeutic resistance that were conferred largely by alterations, not only in the tumor cells, but also in their microenvironment, indicates the importance of taking into account the tumor cell extrinsic compartments. The nature of the vasculature, the presence of cancer associated fibroblasts, the presence/absence of immune cells, the signaling network between tumor cells and stromal cells are the most studied components that could influence treatment response (52).

As previously shown, Dbait administration to mice did not increase the sensitivity of healthy tissue around the tumor to RT (18). In a previous study, we showed that Dbait does not induce cell cycle arrest (18, 20). Hence the specificity of action of Dbait in tumor cells could be due to an impairment in cell cycle checkpoints that is frequently reported in tumors. Tumors cells would be able to divide despite Dbait induced unrepaired breaks and therefore enter mitotic catastrophe. p53 mutations are often associated with this deficient cell cycle controls (18, 53). At the contrary, non-tumor cells with proficient cell cycle control stop dividing until repair is completed, that can take place when Dbait molecules have disappeared (18, 54). Therefore, Dbait, which does not make new lesions on chromosomes but prevents DNA repair of RT induced damage, is toxic for dividing tumor cells but not for healthy tissues. Dbait toxicology studies were realized in wistar rats and cynomolgus monkeys. They showed that the only side effect was a slight to moderate, dose-dependent and reversible inflammatory response at injection sites (18, 55). The tolerance of the clinical form of Dbait (called AsiDNA) in association with RT has been tested in first-in-man phase 1 trial (DRIIM) for patients with in-transit metastases of melanoma (23). No dose-limiting toxicity was observed and the maximum-tolerated dose was not reached.

Oncology has entered an era of personalized medicine in which the selection of treatments for each cancer patient becomes more individualized (56). Identifying predictive biomarkers of treatment sensitivity or resistance is becoming a major challenge. In this study, we have chosen the RPPA technology to search for potential protein biomarkers of Dbait resistance. This technology presents many advantages: it requires only a few micrograms of protein lysate to study activation of cell signaling pathways and allows the comparison of hundreds of samples in the same experiment (10). Thus, we included replicate samples for the cell lines, and different tumor regions of multiple mice for the xenografts. We were able to obtain robust data and assess heterogeneity within and among tumors (10). Here we showed that basal Phospho-H2AX/H2AX and Phospho-NBS1/NBS1 activations [two major actors of DNA damage signaling and cell cycle control (57, 58)] were significantly correlated with Dbait resistance. Interestingly, whereas the amount of phosphorylated H2AX and NBS1 was moderately indicative of sensitivity to Dbait, the frequency of phosphorylated molecules became highly indicative suggesting that resistance is linked to the intensity of chromatin modification. These constitutive activations may reflect that tumor cells are used to survive despite a basal disturbance of DNA damage signaling and cell cycle control and thus are resistant to Dbait. We have previously reported that Dbait molecules disorganize the downstream DNA damage response notably through H2AX and NBS1 disruption (20). In models with a constitutive high level of NBS1 and H2AX activations, low-dose Dbait failed to enhance these disturbances once more and higher concentrations were required. If we compare the results obtained from Dbait-resistant CDX (CB193, SF767 and U87MG) with the ones of the corresponding cell lines, we can note that CB193 and SF797 were also Dbait-resistant *in vitro* while U87MG was Dbait-sensitive. Interestingly, concerning CB193 and SF767 cell lines, they were also the two cell lines harboring the highest level of Phospho-NBS1/NBS1 activation and among the highest level of Phospho-H2AX/H2AX activation. Concerning U87MG, the difference in Dbait response could, at least partly, be explained by the differences in the proteic profiles that can exist between a cell line and its corresponding xenograft. We have previously showed (25) that U87MG had a relatively important difference between *in vitro* and *in vivo* proteic profiles with 37/89 (42%) proteins differentially expressed with a fold change > 1.5.

In this study, we provide the preclinical proof of concept that a combination of RT with Dbait (an inhibitor of DNA repair) could be of interest in the treatment of high grade glioma. A first-in-human phase I trial has evaluated the therapeutic potential of local Dbait injections in combination with RT to treat patients with in-transit metastases of melanoma and provided encouraging results (18, 23). The preclinical data we report suggest that a clinical trial combining HSRT and Dbait could be considered in the treatment of recurrence high grade glioma.

## Data Availability

All datasets generated for this study are included in the manuscript and/or the Supplementary Files.

## Ethics Statement

The Local Committee of Institut Curie on Ethics of Animal Experimentation approved all experiments.

## Author Contributions

JB and EC designed the study, collected, and analyzed the data. They also wrote the manuscript. MD designed the study and carefully revised the manuscript. NB, LdK, and FC have contributed to all experiments. They also gave important intellectual input and carefully revised the manuscript. BP have done statistical analysis and carefully revised the manuscript. PV supervised the study and contributed to data interpretation. All authors approved the final manuscript for submission.

### Conflict of Interest Statement

MD is cofounder of DNA Therapeutics, Onxeo. The remaining authors declare that the research was conducted in the absence of any commercial or financial relationships that could be construed as a potential conflict of interest.
