# Supplementary figures and images for: Combining the DNA Repair Inhibitor Dbait With Radiotherapy for the Treatment of High Grade Glioma: Efficacy and Protein Biomarkers of Resistance in Preclinical Models
Source: Front Oncol. 2019 Jun 19;9:549. doi: 10.3389/fonc.2019.00549 (PMC6593092; doi:10.3389/fonc.2019.00549)

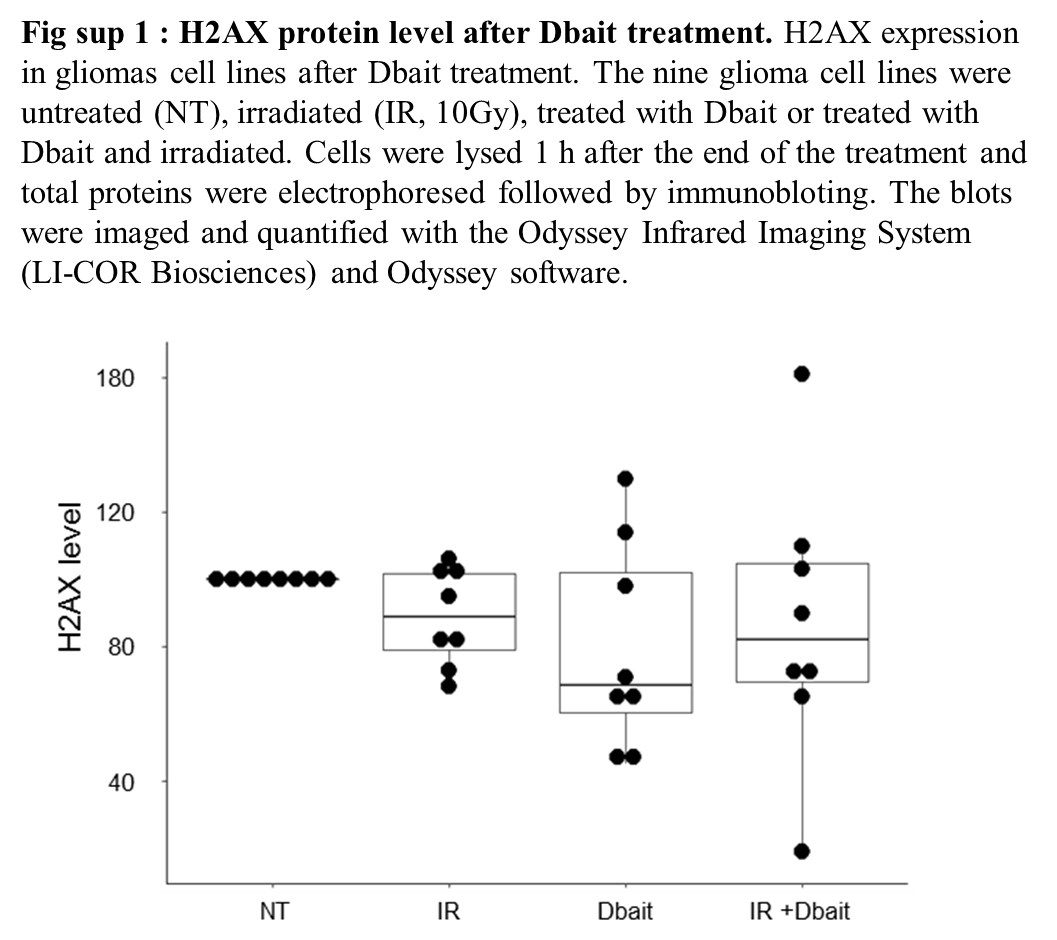

Supplement: Supplementary file 1 [file Image_1.TIF]
