# Supplementary material for: Combining the DNA Repair Inhibitor Dbait With Radiotherapy for the Treatment of High Grade Glioma: Efficacy and Protein Biomarkers of Resistance in Preclinical Models
Source: Front Oncol. 2019 Jun 19;9:549. doi: 10.3389/fonc.2019.00549 (PMC6593092; doi:10.3389/fonc.2019.00549)
Supplement: Supplementary file 2 [file Table_1.doc]

| **Table S.1 : Proteins explored by Reverse Phase Protein Array** | | |
| --- | --- | --- |
| ***Cell survival/Apoptosis*** | ***DNA damage repair*** | ***Stress*** |
| Akt | 53BP1 | COX2 |
| Bcl10 | Ape1 | HSF1 |
| Cleaved PARP (Asp214) p89 | DNA-PKcs | HSP27 |
| EGFR | FEN1 | Hsp90 α |
| FoxO3a/FKHRL1 | Histone H2AX | Hsp90 β |
| Fyn | MGMT | HSPA2/HSP70 |
| HER2/ErbB2 | NBS1 p95 | Phospho-HSP27 (Ser82) |
| IKK α | p53 | Phospho-HSP27 (Ser82) on HSP27 |
| IKK β | Phospho-53BP1 (Ser1778) | Phospho-Hsp90 α (Thr5/7) |
| JNK/SAPK1 | Phospho-DNA-PK (Ser2612) | VCP |
| NF-kB p65 | Phospho-Histone H2AX (Ser139) | Phospho-Hsp90α (Thr5/7) on Hsp90α |
| PARP uncleaved p116 | Phospho-NBS1 p95 (Ser343) |  |
| Phospho-Akt (Ser473) | Phospho-p53 (Ser15) | ***Cell cycle*** |
| Phospho-Akt (Thr308) | Phospho-p53 (Ser392) | Cdc25C |
| Phospho-c-Abl (Tyr204) | Phospho-Topoisomerase II α (Thr1343) | Chk1 |
| Phospho-EGFR (Thr669) | Rad21 | Chk2 (1C12) |
| Phospho-EGFR (Tyr1173) | Phospho-Histone H2AX (Ser139) on H2AX | Phospho-Chk1 (Ser280) |
| Phospho-FoxO3a/FKHRL1 (Thr32) | Phospho-p53 (Ser15) on p53 | Phospho-Chk2 (Thr68) |
| Phospho-JNK/SAPK (Thr183/Tyr185) | Phospho-p53 (Ser392) on p53 | Phospho-Rb (Thr356) |
| Phospho-NF-kB p65 (Ser536) | Phospho-NBS1 p95 (Ser343) on NBS1 | Rb |
| Phospho-p38 MAPK (Thr180/Tyr182) | Phospho-53BP1 (Ser1778) on 53BP1 | Jun B |
| Phospho-PTEN (ser380/Thr382/383) | Phospho-DNA-PK (Ser2612) on DNA-PK | Phospho-JunB |
| PI3 Kinase p110 subunit β |  | Phospho-Rb (Thr356) on Rb |
| PTEN | ***Migration/Invasion*** | Phospho-Chk1 (Ser280) on Chk1 |
| Src | FAK | Phospho-Chk2 (Thr68) on Chk2 |
| Phospho-EGFR (Thr669) on EGFR | Integrin beta4 | Phospho-JunB on JunB |
| Phospho-EGFR (Tyr1173) on EGFR | Phospho-FAK (Tyr861) |  |
| Phospho-Akt (Ser473) on Akt | Phospho-FAK (Tyr925) |  |
| Phospho-Akt (Thr308) on Akt | Phospho-Vimentin (Ser459) |  |
| Phospho-NF-kB p65 (Ser536) on NF-kB | Vimentin |  |
| Phospho-PTEN (ser380/Thr382/383) on PTEN | Phospho-Vimentin (Ser459) on Vimentin |  |
| Cleaved PARP (Asp214) p89 on PARP | Phospho-FAK (Tyr861) on FAK |  |
| Phospho-FoxO3a/FKHRL1 (Thr32) on FoxO3a/FKHRL1 | Phospho-FAK (Tyr925) on FAK |  |
| Phospho-JNK/SAPK (Thr183/Tyr185) on JNK/SAPK |  |  |
